# The role of saliva in gonorrhoea and chlamydia transmission to extragenital sites among men who have sex with men: new insights into transmission

**DOI:** 10.1002/jia2.25354

**Published:** 2019-08-30

**Authors:** Eric PF Chow, Christopher K Fairley

**Affiliations:** ^1^ Melbourne Sexual Health Centre Alfred Health Carlton VIC Australia; ^2^ Central Clinical School Monash University Melbourne VIC Australia

**Keywords:** *Neisseria gonorrhoeae*, *Chlamydia trachomatis*, transmission, men who have sex with men, control, sexual behaviours, sexual practices, saliva, kissing, throat, anal, sexually transmitted infections, sexually transmitted diseases

## Abstract

**Introduction:**

Gonorrhoea and chlamydia cases have been rising among gay, bisexual and other men who have sex with men (MSM) over the last decade. The majority of cases are extragenital and occur at the oropharynx and anorectum. The aim of this narrative review was to review the risk factors and mode of transmission for gonorrhoea and chlamydia at the oropharynx and anorectum among MSM.

**Results and discussion:**

New evidence suggests that oropharyngeal gonorrhoea can be transmitted by kissing in addition to through the established route of condomless oral sex; and anorectal gonorrhoea can be acquired when saliva is used as a lubricant for anal sex and rimming in addition to the established route of condomless penile‐anal sex in MSM. In contrast, condomless penile‐anal sex remains the major route for chlamydia transmission.

**Conclusions:**

Substantial transmission of gonorrhoea may occur with practices other than the established routes of condomless oral and/or anal sex and hence condoms may not be effective in preventing gonorrhoea transmission to extragenital sites. In contrast, condoms are effective for chlamydia control because it is mainly transmitted through condomless penile‐anal sex. Novel interventions for gonorrhoea that reduce the risk of transmission at extragenital site are required.

## 1 Introduction

Sexually transmitted infections (STIs), are increasing globally, particularly in gay, bisexual and other men who have sex with men (MSM) 1, 2, 3, 4, 5. To address these rises, there have been many novel campaigns and interventions for STIs but these interventions have not been associated with effective STI control in MSM 6, 7, 8, 9, 10, 11. The failure of these campaigns and interventions to reduce STIs could be due to several reasons including that they are not reaching the core group, or because they are based on an incomplete understating of how infections are transmitted. The control of STIs may be more difficult with the introduction of treatment as prevention (TasP) and HIV pre‐exposure prophylaxis (PrEP) which has successfully reduced new incident HIV in MSM but has been associated with changes in sexual practices that increase STI risk 12, 13, 14. In this context, it is time to revisit our understanding of how infections are transmitted with the aim of designing new effective interventions to improve STI control.

Both gonorrhoea and chlamydia until recently were thought to be mainly transmitted through condomless penetrative intercourse such as penile‐vaginal, penile‐anal and oral sex 15, 16, 17, 18, 19, 20, 21, 22, 23. However, in the context of rising STI rates and ineffective interventions, it is important to review the transmission of both infections particularly at extragenital sites in MSM which are largely asymptomatic 24, 25, 26, 27. Other anal sexual activities such as fingering, fisting and rimming are commonly practiced among MSM and may play an important role in transmission 28, 29, 30. Several epidemiological studies have found these activities are associated with the acquisition of any STIs (that is, gonorrhoea, chlamydia or syphilis) in MSM; 28, 31, 32 however, there are limited studies examining the role of these practices in the transmission of gonorrhoea and chlamydia independently. The aim of this narrative review was to revisit the transmission of gonorrhoea and chlamydia to the oropharynx and anorectum in MSM. Several reviews have already described the prevalence and epidemiology of gonorrhoea and chlamydia, behavioural and social risk factors, and possible interventions; 1, 4, 17, 33, 34, 35 and thus these areas will not be covered in this review.

## 2 Results and discussion

### 2.1 Prevalence of extragenital gonorrhoea and chlamydia in MSM

Many epidemiological studies have reported on the point prevalence of extragenital gonorrhoea and chlamydia in MSM. Chan and colleagues published a review in 2016 summarising the prevalence of extragenital gonorrhoea and chlamydia from 53 studies (Table 1); 17 however, these estimates vary substantially across geographical regions and study settings. Overall, the authors reported that the median prevalence of gonorrhoea at the oropharynx (4.6%) was similar to the anorectum (5.9%). In contrast, the median prevalence of chlamydia in the anorectum (8.9%) was much higher than in the oropharynx (1.7%).

**Table 1:** Prevalence of extragenital gonorrhoea and chlamydia in MSM

STI	Prevalence (%)	
	Median	Range
Oropharyngeal gonorrhoea	4.6%	0.5% to 16.5%
Oropharyngeal chlamydia	1.7%	0% to 3.6%
Anorectal gonorrhoea	5.9%	0.2% to 24.0%
Anorectal chlamydia	8.9%	2.1% to 23.0%

Note. Data were obtained from a review of 53 studies published by Chan *et al*. (2016) 17.

Several studies have compared the prevalence of extragenital gonorrhoea and chlamydia in MSM by HIV status. A US study reported that HIV‐positive MSM had a higher prevalence of anorectal gonorrhoea (8.2% vs. 3.3%) and anorectal chlamydia (9.0% vs. 6.6%) than HIV‐negative MSM; however, the prevalence was similar in both groups for oropharyngeal infection (that is oropharyngeal gonorrhoea: 5.2% in HIV‐positive vs. 4.3% in HIV‐negative; oropharyngeal chlamydia: 1.6% in HIV‐positive vs. 1.3% in HIV‐negative) 15. These findings are consistent with another study conducted in Melbourne, Australia, showing that the prevalence of anorectal gonorrhoea was higher in HIV‐positive MSM (15.4%) than in HIV‐negative MSM (7.3%) but the prevalence of oropharyngeal gonorrhoea was similar in both in HIV‐positive MSM (9.9%) and HIV‐negative MSM (8.1%) 36.

### 2.2 Oropharyngeal gonorrhoea

Oropharyngeal gonorrhoea is relatively short lived and commonly asymptomatic 7, 25, 37, 38, 39. A natural history study of 18 individuals (12 men and six women) with untreated oropharyngeal gonorrhoea has suggested that the majority of oropharyngeal gonorrhoea infections clear by six weeks and all by 12 weeks 39. Another natural history study of 60 untreated individuals with positive oropharyngeal gonorrhoea culture has shown that more than half (55%) of oropharyngeal gonorrhoea infections clear within seven days 40. Other epidemiological studies also support the short duration of oropharyngeal gonorrhoea 24, 38, 41. However, length time bias may have occurred due to the detection of prevalent infection in these studies.

Oropharyngeal gonorrhoea had been thought to be primarily acquired from oro‐genital contact such as condomless fellatio (Table 2) 24, 42. Fellatio is commonly practiced among MSM (that is, 72.7% of MSM had fellatio with their last male partner) and condoms are rarely used for fellatio in MSM 43, 44.

**Table 2:** Summary of studies examining the route of gonorrhoea and chlamydia transmission to the oropharynx in MSM

Possible route of transmission	Oropharyngeal gonorrhoea	Oropharyngeal chlamydia
Kissing	21, 41, 56, 57	63
Insertive rimming (oral‐anal)	21, 41, 56	63
Fellatio (oral‐penile)	41, 56	63

Studies of symptoms associated with urethral gonorrhoea have been contradictory. In the 1970s, two studies have reported that about 40% of heterosexual men reporting contact with their female partners with gonorrhoea had asymptomatic urethral gonorrhoea 45, 46. However, other studies report that at least 90% of men with urethral gonorrhoea are symptomatic 26, 27, 47, and develop dysuria and urethral discharge within two to five days of exposure 47, 48.

In countries with good access to healthcare, men with symptomatic urethral gonorrhoea usually receive treatment within a few days of the onset of symptoms 49. In this context, the point prevalence of urethral gonorrhoea is estimated to be relatively low in the MSM population (approximately 0.2% 50) compared to the extragenital sites; and hence, this has led some investigators to question whether urethral infection alone could be responsible for the high incidence of oropharyngeal gonorrhoea (26 per 100 person‐years) 50. This is consistent with the observation by Passaro (2018) who reported the prevalence of oropharyngeal gonorrhoea did not differ between MSM who had receptive oral‐penile sex (10.3%) and those who did not (9.8%) 20. Indeed some investigators have hypothesized that the oropharynx may be a more important anatomical site for gonorrhoea transmission in MSM than the urethra 50, 51.

These same investigators have undertaken a series of studies related to their hypothesis. They undertook a study of 33 MSM with untreated culture positive oropharyngeal gonorrhoea and obtained saliva samples from these men up to 14 days after screening 52. The study found that all men (100%) tested positive for *Neisseria gonorrhoeae* in saliva by nucleic acid amplification test (NAAT) and almost half (43%) of men where their saliva samples were detected by culture 52, 53. In addition, two past studies in the 1970s and 1980s have also found that gonorrhoea can be cultured from saliva but the estimates ranged between 8% 54 and 67% 40. Furthermore, these findings raise the question of whether oropharyngeal gonorrhoea could potentially be transmitted between the oropharynges through kissing, and also between the oropharynx and the anorectum through rimming (Table 2) 21, 22, 41, 50.

Several case reports purposed kissing could be a risk factor for oropharyngeal gonorrhoea in the 1970s 37, 54, 55. In these early studies, cases were diagnosed using culture, which has poor sensitivity and specificity for *Neisseria gonorrhoeae* in the oropharynx. Rather surprisingly, there have been only three epidemiological studies conducted since using NAAT, which is a more sensitive test than culture.

The Australian Health In Men (HIM) study by Templeton *et al*. was the first longitudinal study examining the association between oropharyngeal gonorrhoea and kissing 41. The HIM study recruited 1427 HIV‐negative MSM in Sydney between 2001 and 2007 and they found that both dry and wet kissing with casual partners in the last six months were associated with the incident oropharyngeal gonorrhoea. However, this association disappeared after adjusting other sexual practices including receptive condomless fellatio and insertive oro‐anal contact (that is rimming). In the multivariable analysis, men who often engaged in insertive rimming were 1.6 (95% CI: 1.1 to 2.5) times more likely to have oropharyngeal gonorrhoea than men who never engaged in insertive rimming in the last six months.

The second study by Cornelisse *et al*. was an age‐matched 1:2 case–control study conducted among 531 MSM in Melbourne in 2015 56. Similarly, the study found that men who kissed their casual partners in the last three months were 2.2 (95% CI: 1.3 to 3.6) times more likely to have oropharyngeal gonorrhoea than those who did not kiss their casual partners in the univariable analysis. However, the authors were not able to perform multivariable analysis due to high collinearity with other sexual practices. Consistent with Templeton *et al*.'s study, Cornelisse *et al*.'s study also identified that both insertive rimming and receptive fellatio are risk factors for oropharyngeal gonorrhoea in MSM in the univariable analysis 41.

Both Templeton *et al*.'s 41 and Cornelisse *et al*.'s study 56 measured kissing as part of sexual practices and did not investigate kissing without sex as a risk factor. The third study by Chow *et al*. addressed this concern 57. It was a cross‐sectional study conducted among 3677 MSM in Melbourne in 2016‐2017 that measured male partners in three different categories: (1) kissing‐only partners where men only kissed their partners but did not have sex with them; (2) sex‐only partners where men only had sex with their partners but did not kiss them; and (3) kissing‐with‐sex partners where men kissed and had sex with their partners. Chow *et al*.'s study defined sex as any oral or anal sexual contacts and the finding showed that both kissing‐only and kissing‐with‐sex partners in the last three months were strongly associated with oropharyngeal gonorrhoea in the adjusted analysis. In addition, the risk of oropharyngeal gonorrhoea increased with an increasing number of kissing‐only and kissing‐with‐sex partners. However, the number of sex‐only partners in the last three months was not associated with oropharyngeal gonorrhoea. This was the first study identified to show kissing in the absence of sex may be an important and neglected risk factor for oropharyngeal gonorrhoea; however, this study did not measure oral sex as a separate sexual act and so could not adjust for it separately.

Although studies have shown that kissing may be a risk factor for oropharyngeal gonorrhoea in MSM, the role of saliva in gonorrhoea transmission is still poorly understood. If saliva can carry infectious gonorrhoea, it is hypothesized that men could acquire oropharyngeal gonorrhoea through kissing by contacting infectious saliva, but it is unclear how much saliva is adequate for gonorrhoea transmission. Moreover, the salivary flow and its production vary between individuals. It is estimated that the salivary flow rate is about 0.3‐0.4 ml per min while unstimulated 58, and the salivary flow rate increases up to 4‐5 ml per min while stimulated such as chewing and eating but no studies have assessed saliva flow rates during kissing or sex 59, 60.

### 2.3 Oropharyngeal chlamydia

The majority of the oropharyngeal chlamydia infections are asymptomatic in men and therefore their diagnosis primarily depends on asymptomatic screening 61, 62. Unlike oropharyngeal gonorrhoea, age does not seem to be a significant predictor for oropharyngeal chlamydia 62, 63. The HIM Study was a longitudinal study examining the risk factors for oropharyngeal chlamydia transmission in MSM. 63 The HIM study found that men who often engaged in receptive oral‐penile sex with ejaculation were 5.3 (95% CI: 1.7 to 16.7) times more likely to have oropharyngeal chlamydia compared to men who never had receptive penile‐oral sex with ejaculation with their casual partners in the last six months (Table 2) 63. Other activities (for example kissing (both dry and wet kissing), receptive oral‐penile sex without ejaculation and insertive rimming) have found to be not associated with oropharyngeal chlamydia among MSM (Table 2) 63. Similarly, a study conducted in Lima, Peru has shown that there was no significant difference in the prevalence of oropharyngeal chlamydia between MSM who had receptive oral‐penile sex (4.1%) and those who did not (3.4%) 20. However, a strong association between oropharyngeal chlamydia acquisition and history of receptive oral‐penile sex was observed among women 64.

A number of laboratory studies have been undertaken to examine the role of saliva in oropharyngeal chlamydia transmission. Two studies in the 1990s found that saliva has an inhibitory effect against *Chlamydia trachomatis* 65, 66. Further studies with better technology and a more sensitive diagnostic test are important to validate whether saliva can carry chlamydia to provide a better understanding of transmission.

### 2.4 Anorectal gonorrhoea

Most men infected with gonorrhoea in the anorectum are asymptomatic; 27 however, among those with symptoms, anal discharge, pain and itching are common. Similar to oropharyngeal gonorrhoea, younger MSM are at higher risk of acquiring anorectal gonorrhoea than older MSM 67, 68. Condomless anal sex is a clear risk factor for anorectal gonorrhoea 67, 69, 70. But other modes of transmission may also occur. For example, an epidemiological study has found that there was no difference in the prevalence of anorectal gonorrhoea between MSM who had receptive penile‐anal sex (8.8%) and those who did not (6.6%) 20. This suggests that other non‐receptive anal sexual intercourses (for example, receptive fingering, receptive fisting (insertion of the hand into the rectum), receptive rimming and dildo insertion) may also be associated with anorectal gonorrhoea (Table 3) 67.

**Table 3:** Summary of studies examining the route of gonorrhoea and chlamydia transmission to the anorectum in MSM

Possible route of transmission	Anorectal gonorrhoea	Anorectal chlamydia
Oral infection passing through the gastrointestinal tract to the rectum	Nil	73, 75, 76
Receptive rimming (oral‐anal)	30, 67	67, 72
Receptive fisting	67	67
Receptive fingering	[30, 67]	67, 72
Receptive of dildos	67	67
Saliva use as a lubricate for anal sex	30	72

Given that saliva can carry infectious gonorrhoea, it is hypothesised anal sex that involves in saliva (for example use of saliva as a lubricant for anal sex or saliva on a penis before insertion) may be associated with anorectal gonorrhoea (Table 3). A cross‐sectional study of 283 young MSM conducted in San Francisco has shown that about 87% of MSM used saliva as a lubricant for anal sex during their lifetime but only 31% of MSM used it in the last six months 71. Another study conducted among 1312 MSM in Melbourne in 2014‐2015 found that 69% of MSM used saliva as a lubricant for anal sex in the last three months 30. The authors also identified that men who used saliva as a lubricant for anal sex were 2.2 (95% CI: 1.0 to 4.7) times more likely to have anorectal gonorrhoea by culture than those who did not use saliva as a lubricant for anal sex after adjusting for other confounding factors including condom use.

### 2.5 Anorectal chlamydia

Anorectal chlamydia is primarily transmitted through condomless penile‐anal sex in MSM 22, 67. The HIM study found that receptive fingering, receptive fisting and receptive rimming were risk factors for incident anorectal chlamydia in the univaraible analysis; however, only receptive fingering was an independent risk factor after adjusting for other confounding factors (Table 3) 67. The authors concluded the men who often had receptive fingering were 4.6 (95% CI: 2.3 to 9.3) times more likely to have anorectal chlamydia than those who never had receptive fingering in the last six months. Unlike anorectal gonorrhoea, a Melbourne study by Cornelisse *et al*. showed that the use of saliva as a lubricant for anal sex is not a risk factor for anorectal chlamydia in MSM 72.

A meta‐analysis published in 2019 has concluded that anal intercourse is associated with anorectal gonorrhoea but not with anorectal chlamydia among women 16. This suggests that the mode of transmission for gonorrhoea and chlamydia is likely to be different and hence it leads to several new hypotheses of anorectal chlamydia acquisition. Animal studies have shown that chlamydia can survive in the gastrointestinal tract suggesting that this may also apply to human 73, 74. New paradigm has been proposed that it is possible oropharyngeal chlamydial infection can pass through the gastrointestinal tract to the anorectum 73, 75, 76. However, further studies are certainly required to confirm this hypothesis.

## 3 Conclusions

MSM can acquire gonorrhoea and/or chlamydia at the oropharynx and anorectum although the epidemiological evidence suggests the modes of transmission differ. For gonorrhoea, infections at extragenital sites are transmitted through non‐genital contacts such as kissing, rimming and use of saliva in addition to condomless oral or anal sex. For chlamydia, condomless anal sex is the main risk factor. However, the uncertainty about the hypotheses of the route of transmission for gonorrhoea and chlamydia via saliva among MSM should be acknowledged 77. This uncertainty arises in part because infection at multiple sites is common in MSM and multiple sexual practices usually occur during one single sex act 20, 43, making it difficult to clearly delineate which specific sexual practice was responsible for the transmission between anatomical sites 77. Furthermore, existing data regarding chlamydia transmission to extragenital sites are insufficient to draw meaningful conclusions and thus more research is required. Condoms may not necessarily be effective in preventing some extragenital infections 78. Other interventions that target the extragenital site related to its mode of transmission are required 1, 35, 79, 80.

## Competing interests

EPFC has received consultant fees from Gilead Sciences and speaker's fee from Seqirus Australia and research grants from Merck Sharp & Dohme outside the submitted work. CKF owns shares in CSL limited which markets Gardasil in Australia.

## Authors’ contributions

EPFC wrote the first draft of the manuscript. All authors contributed to the concept, literature search, writing and revisions of the manuscript. All authors critically revised it for important intellectual content and approved the final version of the manuscript.
